# Supplementary material for: PyRates—A code-generation tool for modeling dynamical systems in biology and beyond
Source: PLoS Comput Biol. 2023 Dec 27;19(12):e1011761. doi: 10.1371/journal.pcbi.1011761 (PMC10775981; doi:10.1371/journal.pcbi.1011761)
Supplement: Attachment — Submitted filename: rebuttal.pdf [file pcbi.1011761.s003.pdf]

Dear Editor,

We would like to thank you for the opportunity to resubmit our manuscript “PyRates - A Code-Generation Tool for Modeling Dynamical Systems in Biology and Beyond”, with revisions. We are confident that we have addressed all concerns and questions raised by the referees.

Most importantly, we added a new use example of our software to the Software Structure section, which explains the model implementation language of PyRates via the example of a classic predator-prey model. We further provided the model definition files for all dynamical systems models that we make use of in the manuscript as supplementary material and added a concrete example of how our software supports access to dynamical systems analysis tools in different programming languages.

We provide a detailed point-by-point response to the reviewer’s comments below.

Yours sincerely,

Richard Gast, Thomas R. Knoesche, and Ann Kennedy

Responses to Reviewer #1:

The manuscript presents a python-based software package for implementing dynamical models of ordinary- and delay-differential equations for multiple target platforms. It uses a novel modelling language to generate code for running the model on existing solvers in Python, Fortran, Matlab or Julia. This gives users the flexibility to choose the most appropriate solver for their model without altering the definition. The manuscript is well written and the ability of the software to generate code for multiple back-ends is beneficial. Although it is worth mentioning cellML (www.cellml.org) as another software package with a similar aim.

We thank the reviewer for the positive evaluation of our manuscript and software. We added a reference to cellML in the section of the manuscript where we compare PyRates to other dynamical systems tools (line 97).

I would have liked the manuscript to begin with an example of the model definition language, say for the Van der Pol equation, before going on to describe the software structure. As it stands, the examples only demonstrate how to re-use the existing model definitions. There are no examples of how to define a new set of equations from scratch, which is where I would expect to start as a new user. Nonetheless, the current examples do provide compelling evidence of the applicability of the software to a diverse set of numerical tools across multiple programming languages.

This is a good suggestion, and we have added a use example to the frontend section that introduces the model definition language using the Lotka-Volterra equations for predator-prey

population dynamics. This example explains the model definition via the Python user interface, introducing the core concepts of Operator, Circuit, Node, and Edge Templates. For all other use examples, we now provide the full YAML definition files as supplementary material, allowing the reader to explore each model definition as it is constructed from scratch.

We also direct readers to our documentation website for a more in-depth description of the model definition language, mathematical formalism, and various options that users can choose from when creating a model (https://pyrates.readthedocs.io/en/latest/). This documentation covers many implementation details that we do not discuss in the manuscript, since it would lead to a considerable increase in its length. Rather than document all features of PyRates in this manuscript, we see this manuscript as a way to (i) make the PLOS CB readership aware of our software, (ii) provide a comprehensive overview of the software structure, scope, and its capabilities, and (iii) provide a peer-reviewed article that can be referenced by future users of the software.

The software makes it easy to build hierarchical models by coupling existing subsytems (operator nodes) together using operator edges. It is tempting to assume that ever larger models could be assembled using this approach. However, large hierarchical models are ultimately limited by the solver needing to accommodate the subsystem with the fastest timescale. So even though hierarchical models do have clear benefits of rapid-prototyping and code re-use, it is worth emphasizing that good model design is still needed to avoid wasting time computing the dynamics of subsystems that may have negligible impact on the global dynamics.

We fully agree with the reviewer: a disadvantage of hierarchical models with substantial differences in the time scales is that the system can be dominated by the dynamics of the slowest subsystem, while still requiring simulation with a small time step to capture the behavior of the fastest subsystem. This is a general limitation that choice of simulation software can't fully overcome, though as the reviewer points out it can be avoided with careful model design. Also, if the scientific question is to find out under which conditions the detailed description of a fast, low-level subsystem may be negligible for the global model dynamics, (ie under which conditions fast subsystems may be approximated by their steady-state solutions), it is still relevant to study such models, and PyRates provides one easy way to do so. We added a sentence to the manuscript that reflects the points the reviewer raises (lines 212-220).

Minor Comments

p6. Line 144: I was confused by the example of the operator in the context of a neural population. Surely, the output (dy/dt) is the instantaneous change of the input (y). The units of synaptic current (input) and firing rate (output) in your example do not follow that rule.

Thank you for pointing out the misleading formulation in that sentence. Indeed, the change of units from current to firing rate makes the neural population a bad first example for the reader. In response to your comment, we have changed the example we use to describe the operator

template, and now use the Lotka-Volterra equations, in which the input is a population density and the output is a population density as well, which should be less confusing.

Just to provide an explanation for the current/firing rate point - PyRates supports the construction of operators with mismatched input and output units. For example in a leaky-integrator neuron, the input is a synaptic current, which leads to a change in the membrane potential of the neuron, which is translated into an output firing rate via a sigmoidal activation function. In the actual biological system, this firing rate reflects a series of action potentials that induce a synaptic current input in the postsynaptic neuron, completing the cycle.

p6. I felt myself wanting a little more detail about using edges to combine operator templates (nodes) using edges. Specifically, how do the outputs of some nodes become the inputs to others? An example might help.

To address this comment, we added the Lotka-Volterra model as an example, which includes the explicit definition of an operator template with an input and output variable, and the subsequent coupling of these variables via edges (see frontend section). In addition, we refer to the readthedocs use example for more details on the template definition system. On readthedocs, we provide a detailed introduction on how operators within and across nodes can be coupled with each other (see https://pyrates.readthedocs.io/en/latest/auto_implementations/yaml_definitions.html for example).

p8. Line 244: It would be useful to see an example of the mode definition before coupling them together with the circuit template.

We have now added the full YAML definition files for all use examples as supplementary material.

p11. As I understand it, the Quadratic Integrate and Fire (QIF) model involves a reset when the voltage exceeds a threshold. Yet the manuscript states (Line 580) that PyRates does not support event handling like that. Can you please clarify the apparent contradiction?

We apologize for this confusion.The QIF model that comes pre-implemented in PyRates, and that we made use of in this manuscript, is a mean-field model of a population of QIF neurons, i.e. it models the dynamics of smooth macroscopic variables such as the population-average firing rate and membrane potential. While those mean-field equations have been derived from a network of spiking neurons (see Gast et al. 2020 Neural Computation), they are self-consistent and thus do not require solving the single neuron equations that contain the resetting mechanism. We clarified this in the revised manuscript (lines 425-436).

We also note that while PyRates does not support handling of spike resets, the PyRates module RectiPy does, allowing our system to still be used for spiking network simulations.

<u>Responses to Reviewer #2:</u>

Originality: the manuscript states that there are a variety of tools serving similar purpose. A unique point for the presented library is that it can serve as an interface to multiple "backends". That is, one formulates or imports the model into pyrates once and then can use several numerical engines to perform different types of mathematical analysis.

Beyond that, for intricate mathematical analysis tools such as Auto-07p the initial provision of model files (e.g. in FORTRAN) poses a significant barrier to entry for practicioners in the life sciences. The pyrates interface would make these undeservedly obscure tools accessible to a wider community.

Innovation: the tools described in the manuscript provide "glue", linking existing high-level analysis methods (bifurcation analysis, optimization, parallelized parameter studies) to easy-to-create model descriptions and a familiar interface. So, while they may not provide innovative methods in themselves, they make innovative methods accessible to a wider audience.

Importance: this would have to be measured by the level of adoption in the future, but as a developer/maintainer of mathematical bifurcation analysis tools, I am keen to have these interfaces available.

While the software discussed has the potential to support future significant biological and methodological insights and conclusions these criteria are not fully applicable to a manuscript describing a new software tool.

All demonstrations presented in the article are downloadable from an open-source repository.

In summary, I think that PLOSCOMPBIOL is a good audience for the manuscript.

Answer: We would like to thank the reviewer for the detailed, well-structured, and positive evaluation of our manuscript and software.

Comments

1) The introduction (line 85ff) mentions dde-biftool as an example of a backend. Also further down support for bifurcation analysis for systems with delay is mentioned, but neither the article nor the webpage https://pyrates.readthedocs.io/en/latest have any demonstration that I could find. I would be interested to see how this works (it would require interfacing with octave or matlab). If this backend is not yet available then it should not be shown in the table or advertised in the intro as "done", but rather as something that could be supported (in the future, or if specific interest arises).

We first note that we have clarified the language of our revised manuscript regarding our usage of the term “backend”: a PyRates backend is a code-generation module that outputs code for the user to run on a specific third-party platform. In this clarified wording, DDE-BIFTOOL is not a backend itself, but an example of a dynamical systems tool that PyRates provides an interface to via its Matlab code-generation backend. To clarify this, we have altered the text in the introduction where we reference Table 1, and also changed the title and column labeling of Table1.

We have also added a new use example that shows how PyRates can be used to generate model equations for DDE-BIFTOOL. This use example primarily demonstrates the construction of PyRates model definitions, using the example of a Lotka-Volterra equation system with delay-coupling (see section “The PyRates frontend”). In the subsequent section “the PyRates backend”, we demonstrate how the Matlab backend of PyRates can be used to generate a forward function for this example model, which follows the DDE-BIFTOOL syntax for specifying delayed differential equation models. We provided the generated Matlab function file in the supplementary material. The generated function can be used as the user-provided forward function of a model in DDE-BIFTOOL

It would certainly be appealing to write a package which allows to interface DDE-BIFTOOL from within Python. Such a package would be similar to PyCoBi, in that it would use PyRates as a frontend to translate model definitions into DDE-BIFTOOL-compatible run functions, and then provides methods that call Matlab in the background. (PyCoBi takes such an approach to provide a Python interface to the Fortran-based continuation and bifurcation package AUTO: it generates files for Auto-07p, then calls the software in the background.)

Thus, while direct interface to DDE-BIFTOOL from Python is not currently part of PyRates, we list DDE-BIFTOOL as a supported package because it can be used via the PyRates code-generation backend. The manuscript also demonstrates that PyRates can be extended to serve as a powerful frontend for direct interface with dynamical systems tools from within Python, using as example two additional pieces of software, for which we did exactly that: RectiPy and PyCoBi. While it is feasible to create a similar package for direct interface to DDE-BIFTOOL, the purpose of this manuscript is simply to establish the “indirect” approach offered by the PyRates code-generation backend, and to demonstrate the principles by which a direct interface to a third-party tool can be built.

Finally, we would like to note two examples in the online documentation that show how delayed differential equation features of PyRates can be used. The use example at https://pyrates.readthedocs.io/en/latest/auto_implementations/delay_coupling.html shows how to implement various forms of delay coupling, whereas https://pyrates.readthedocs.io/en/latest/auto_analysis/dde.html demonstrates the simulation of a DDE system using the Python software ddeint. By choosing the Matlab backend instead of the Python backend (accomplished by specifying `backend=”matlab”` in the `get_run_func()` call), the same example could be used to generate a Matlab function file that is compatible with DDE-BIFTOOL.

2) The section with the Frontend description uses specific technical terms without justification, eg, "operator template" or "circuit template". These are never explained even though these look like some of the key concepts behind pyrates. For example, "circuit template" appears to be a way to define a (recursive?) graph where nodes are either themselves again circuit templates or operator templates. Edges appear to be defined by tuples. Some of the tuples' entries can be guessed ("source node label", "target node label") others are hard to guess. In particular, the graph appears to be directed by construction, unless one defines all edges in a symmetric fashion.

We apologize for the confusion: while this information was originally omitted to avoid redundancy with the online documentation and our previous manuscript (Gast et al., PloS One 2019), we have now included it to make this paper more independent of these other references. Specifically, we have addressed this comment by adding an example to the frontend section, which introduces the different template classes via the Lotka-Volterra equations with delay-coupling. The introduction is brief and focuses on the most important terms, since in-depth descriptions of the model definition interface are available on our documentation website. However, we do now make clear how edges are defined and that they have to be directed, and how a circuit is constructed from nodes and edges, which are in turn defined via operators.
As the reviewer guessed, circuit templates can be used “recursively”, by reusing them as nodes for higher-level circuits. A brief description of this is available at https://pyrates.readthedocs.io/en/latest/template_specification.html, which we also refer to in the frontend section of the manuscript.

3) Similarly, in Listing 1: conventions are not explained. Later it is mentioned that strings such as 'li_op/r_in' are defined in the YAML templates. At the moment some pieces of the listing hand in the air such that can only speculate about their meaning when reading the manuscript.

We now explain the convention used for specifying model variables as a part of the new use example in the frontend section of the revised manuscript. These conventions also apply to Listing 4 in the revised manuscript (former Listing 1).

4) Comparatively, Listing 2 is better, but, for example, we have "method='DOP853'" in the listing, whereas in the text we have "a Runge-Kutta algorithm of order 8". This way of paraphrasing obscures, obscuring that the text is describing the listing (as it should). Similar instances occur for other listings.

To address this comment, we adjusted the description of Listing 5 of the revised manuscript (former Listing 2). We clarified which part of the listing we refer to when we described the numerical solver in the text (lines 389-391), and added a reference to an online example which describes the grid_search function in more detail. The incorporation of the Lotka-Volterra use example and the description of the template classes should also resolve the other instances where it was not clear what the text was referring to.

5) Figure 3a cannot have been produced by the snippet shown in Listing 5. The reference also points to [6] (a standard review), not the underlying code reproducing Figure 3a in the repository.

We clarified in the manuscript that the snippet in Listing 8 (former Listing 5) merely serves to find the initial steady-state solution from which to start the bifurcation analysis (lines 465-469). We also added a reference in the text to code in the repository https://github.com/pyrates-neuroscience/use_examples that directly reproduces Fig. 3a (lines 507-509).

Response to Reviewer #3:

The submission by Gast et al reports on the authors' work developing a suite of Python-based software libraries aimed at simplifying and enhancing mathematical modelling research in neurobiology and other scientific fields. The principal library is PyRates (so-named in reference to its original intended scope of rate-based neuron and neural population models), an earlier version of which was described in a previous methods paper (Gast et al. PLoS One 2019). The present paper focuses on several major updates to the library since then, including: i) a [nominally] broader scope, up from from "rate-based neural models" to "dynamical systems in biology"; ii) a broader suite of code generation options and languages, and a greater emphasis on code generation per se; iii) two additional supporting libraries, PyCoBI (for parameter continuation) and RectiPy (for parameter optimization), that extend the functionality of PyRates considerably.

At a technical level, the principal innovation of PyRates is its approach to the 'frontend' problem of flexibly and efficiently specifying differential equation models via a hierarchical templating system, and their subsequent translation into 'backend' executable code in a range of programming languages. The template system consists of an 'operator' template and a 'circuit' template, that succeeds in capturing succinctly the essential structure of network models used in neuroscience and other fields. A particularly nice touch at the template parsing stage is the use of SymPy to extract symbolic graphs from the text-specified system equations., which then form the basis of the backend code generation process.

The currently supported backend implementation options are NumPy, TensorFlow, PyTorch, Fortran, Julia, and Matlab. Being able to switch easily between each of these implementation options has several clear advantages. For example, working with the NumPy and Matlab backends may be optimal for understanding, debugging, etc., as familiarity with these languages is more common than the others, in computational neuroscience at least. If execution speed is of priority, then the Fortran or Julia backends are likely to be preferable. If model optimization / parameter fitting is a requirement, then the PyTorch or TensorFlow backends may be preferred. This flexibility is an important feature of the library, although as noted below there is not much in the way of quantitative comparisons between these alternative backend options offered in the manuscript.

After a short technical description of the PyRates frontend and backend architectures and model definition interface, the paper goes on to present usage examples with corresponding code blocks for i) numerical simulations and parameter sweeps, ii) bifurcation analysis, and iii) parameter optimization, respectively using Van der Pol oscillator, quadratic integrate-and-fire, and delay-coupled leaky integrator models of neural dynamics. These and other examples are provided clearly and replicably on the software's documentation website. From my own testing, I have found all of the code in the manuscript and the documentation pages to run without error and as expected.

Overall, the article is well written and compelling, both in its motivation and its example cases. The PyRates library does indeed provide a fairly unique alternative to many of the more established computational neuroscience, with several advantages that the authors describe clearly and succinctly. As is always the case, the most important factors for medium/long-term success of the project will be good documentation, community adoption, continual development, and (eventually) easily replicable high-profile published use cases. The present paper, along with its 2019 predecessor, provide a solid foundation for all of these.

I am therefore happy to recommend the paper for publication in PLoS CB, provided that the authors address the following points:

We would like to thank the reviewer for the detailed and positive evaluation of our manuscript. We appreciate the time spent to test the provided use example code and to understand the architecture of the software.

Major Comments

Distinction from previous work

The authors' previous methodological article on PyRates is mentioned three times in the manuscript:

Line 28: "PyRates was previously developed as a toolbox for neural network modelling [20]".

Line 53: "We then present the software structure of PyRates in detail, noting novel features that have been added since our previous manuscript[20]".

Line 130: "A detailed description of this template-based user interface is provided in our previous work [20], as well as in our online documentation"

Now, despite the presence of these lines, I am still a little concerned that the point of this article being an update on the previous one, and not a primary reference, is not made sufficiently clear.

Given the amount of overlap in content with Gast et al. (2019), I think the authors need to do a little bit more to help communicate that this is the case, and what exactly motivates a follow-up article such as this one.

Specifically, for example,

- There is no mention of the authors' previous work in the Abstract, Author Summary, or the entirety of the Discussion sections. Please fix this.

- Please adjust phrases like 'we introduce', for the above reasons.

- Where previous work is mentioned (eg line 29), please add some more sentences saying what was actually done previously, and how specifically that functionality is now being extended/augmented by the new library updates

We appreciate the feedback of the reviewer on this issue. To address the concerns, we added clarifications regarding the relationship between the current manuscript and the 2019 publication to the author summary as well as the Availability and Future Directions (former Discussion) section (lines 658-669). We also replaced phrases like "we introduce…" with statements like "we present …".

Furthermore, we added text to make explicit the changes that we have made to the model definition system since 2019, and we explain the range of new features that have been added since 2019 (lines 29-41). We believe that these edits address the reviewer's concern regarding acknowledgement of the 2019 manuscript, while also making clearer the many changes to PyRates's scope and functionality that we believe merit this new publication.

We would like to emphasize that the scope and functionalities of PyRates have changed a lot since the initial release in 2019. Of course there are many incremental changes, such as the changes to the model definition language, which supports a much larger range of models to be defined in PyRates (the most important addition being the entire family of delayed differential equations). But more importantly, *the purpose of PyRates is no longer to serve as a simulation tool*. While it still fully supports this functionality, its core function is code generation. It allows users to implement dynamical systems models in a simple, intuitive way, then translate these models into automatically written code for various backends. PyRates's code generation is therefore helpful to end users who wish to study a system using multiple dynamical systems analysis packages written in multiple languages, without the need to reimplement their models in each of these languages. Since differential equations for complex biological systems can become fairly high dimensional, this erases a time consuming and error prone process for modelers.

PyRates is also a valuable resource for tool developers, as we demonstrate with our two new example modules RectiPy and PyCoBi. Using PyRates as a frontend for these tools allowed us to build custom libraries that can perform parameter optimization and bifurcation analysis,

respectively, for any model that can be defined in PyRates. In doing so we did not have to come up with a new user interface for model definitions, since we could just reuse the one provided by PyRates. Used in this way, PyRates improves the software development process for such tools considerably.

Performance comparison of several backends

A key feature of the library is its ability to generate code in multiple scientific programming languages. One of the advantages of this would be to take advantage of the well known faster execution of some languages than others. However, the manuscript does not show any examples of this in action. It would be helpful to see some simple benchmark tests where the same model is run for all of the backend options, and run time is tracked. One would expect an order of something like Fortran, Julia, Numpy, PyTorch, Tensorflow, Matlab. The exact order and how much daylight there is between different languages is of course liable to vary considerably from application to application; although if there is a dramatic and consistent deviation from expectations here that would need to be clearly explained, as it would raise questions around the code generation approach. Secondary optimizations such as vectorization and jit compilation (as mentioned in the paper already) could be incorporated or left out, as the authors see fit.

While we absolutely understand and share the curiosity of the reviewer for how the different backends compare against each other in terms of compute speed, we think that addressing this question properly is out of the scope of this manuscript. The issue we see is the following: each of the backends can be called directly from within Python (where PyRates runs), but here the Python-based backends will have a considerable advantage since they won't require the translation of a Python variable into a variable of another programming language. Thus, the result will be that NumPy, Tensorflow and PyTorch are faster backends than the Fortran, Matlab, and Julia backends if we decided to run the benchmark comparisons all via the "pyrates.CircuitTemplate.run" method (we have observed these run time differences on various examples that we have tried out while developing the software). This comparison would only hold for the highly unlikely use case that a user wanted to use the numerical solver implemented within PyRates, using a very particular, potentially non-Python backend.

The other possibility to run this benchmark comparison is to skip calling the solvers through Python and run them directly in each respective programming language. This can be done either via dedicated wrappers such as PyCoBi and RectiPy (the former solves the issue of unfavorable run times due to variable conversions at run time), or by using the run function generated by PyRates directly in the other programming language. However, at this point, the benchmark comparison is not specific to PyRates anymore, since we have to compare scripts in 4 different programming languages that may involve other tools such as NumPy, Numba, DifferentialEquations.jl, Matlab's ODE solvers, etc.

In this latter, more common PyRates use case, we would not be simply comparing each language as a simulation environment, we would be comparing specific third-party tools written for each language (e.g. DifferentialEquations.jl in Julia or DDE-BIFTOOL in Matlab). These are specialized tools, and it is not trivial to define a single computational task that could be tested in each potential interface. Furthermore, as third-party software, the performances of these packages is out of our control, will depend on the task at hand, and will change as computing resources and the tools themselves evolve.Since the main purpose of this manuscript is to describe new PyRates functionality that has been developed since the initial release in 2019, we respectfully argue that benchmarking third-party backends would be out of scope for this particular work.

Minor Comments

Scope of library

A notable shift in this paper relative to the 2019 one is its generalization of PyRates' scope, from a tool accommodating a specific type of neural modelling, to one supporting dynamical systems modelling in general. All well and good, but it then comes as a bit of surprise that all of the examples shown continue to be of rate-base neural models. The broader and bolder scope that the authors are apparently aiming for would be a bit more convincing if they included examples from some other fields, such as population ecology or kinesiology.

This is a great point, and we agree that it is important to make clear to the reader that PyRates is not specifically a neural modeling tool. To help make this case, the first example of the revised manuscript now demonstrates how the template system of PyRates works using the Lotka-Volterra equations that model predator-prey dynamics in ecology. We must also note that the oscillators used in example 1 and the leaky integrators used in example 3 are not specific to neuroscience: the Van der Pol oscillator was originally proposed as a model of the heart, and the Kuramoto oscillator was developed to model periodic processes of chemical reactions. Furthermore, leaky integrator dynamics can be found in classical models of population dynamics, classic physics models, as well as in cognitive models of perceptual processes (see Ossmy et al. 2013 Current Biology for example). It speaks to the fundamental nature of dynamical systems models that the use cases we showcase in the manuscript have been widely applied to capture the behavior of diverse physical processes. We hope that the adjusted use cases help to demonstrate that none of the functionality of PyRates's graph-based dynamical systems framework is specific to neuroscience applications.

Graphs

Line 211 - The PyTorch graph is mentioned several times in this paragraph. Previously, the SymPy model graph has been mentioned. Please say a few words about the relationships between the two (e.g., SymPy graph is a subgraph of the PyTorch graph ?)

We apologize for this confusion, and have now clarified that the SymPy graph is a backend-independent representation of the system variables and equations, whereas the PyTorch graph is specific to the PyTorch backend and represents the graph called during run-time for forward and backward passes through the graph (lines 288-293).

Run times

Line 267 states “To minimize the runtime of this problem, we use the function pyrates.grid_search, which takes a set of multiple model parameterizations and performs the numerical integration in a single combined model by vectorizing the model equations.”
Can the authors please provide a demonstration that this approach reduces the run time more effectively than comparable alternatives, eg multiprocessing/multithreading with Joblib?

We share the interest of the reviewer in finding and using the most efficient multiprocessing solutions. We would like to emphasize, however, that PyRates (and `grid_search` in particular) has not been optimized to compete with other multiprocessing frameworks. The function `grid_search` is provided as an option to evaluate the model dynamics for multiple model parameterizations in parallel, and within PyRates. To this end, `grid_search` creates multiple copies of a model, combines them into a single, vectorized model, and thus exploits the optimizations that come for vectorized code in a given PyRates backend. For example, the function `grid_search` could be used together with the PyTorch backend to run multiple parameterization in parallel on a GPU, but it could also be used together with the numpy backend and numba-based runtime optimization on CPUs.
Ultimately, the comparison between `grid_search` and multithreading/multiprocessing tools would come down to the following question: When is it more efficient to vectorize code and run it in a single process, as compared to using multiprocessing? The answer to this question will depend on various factors, such as the problem/application that is considered, and the choice of the backend in PyRates. Since computation speed is not a main argument that we raise for using our software (see our response to comment “Scope of the library”), we think that running this type of comparison is out of scope for this particular manuscript.

Templates

The paper would be a little more complete if a full model template is provided. Currently all of the examples shown use pre-defined templates, the contents of which are indicated only schematically in Figure 1A. As such the operator/circuit/template framework, which is a major feature of the PyRates USP, is not demonstrated as fully as it ideally would be.

Please could the authors add an additional figure / listing for one of the model examples that shows one of the templates in full.

To address this comment, we provided a full model template for a predator-prey model in the frontend section. We introduce the different template classes via this example model and also showcase how the templates are translated into a run file via the backend. Furthermore, we added the YAML definition files for all use examples as supplementary material.

Auto-07p

For the benefit of interested readers who are not familiar with the methodology, please provide a few further sentences describing how Auto-07p performs automated bifurcation analysis. (suggest from around line 399 "Starting from this steady-state solution, we can perform parameter continuations and automated bifurcation analysis [6] via PyCoBi.")

Addressed as suggested (lines 488-503).

Typographical / grammatical errors
======================

Line 556 - 'see section '
(no section name/number given)

Addressed by removing the reference.

Line 552-554 -
Inconsistent switching between present and past tense

Addressed.

[FYI: In terms of English language usage, this is one of the most tightly written and least error-strewn manuscripts I have ever reviewed. Good job.]

Thank you!
